# The Potential of Chicken–Herb Essence to Improve Milk Production and Infant Health in the Sprague Dawley Animal Model

**DOI:** 10.3390/foods13111603

**Published:** 2024-05-22

**Authors:** Erna Puspasari, Ahmad Sulaeman, Eny Palupi, Fachriyan Hasmi Pasaribu, Astari Apriantini

**Affiliations:** 1 Department of Community Nutrition, Faculty of Human Ecology, IPB University, Bogor 16680, Indonesia; rnha.puspa@gmail.com (E.P.); enypalupi@apps.ipb.ac.id (E.P.); 2 Department of Animal Diseases and Veterinary Health, Faculty of Veterinary Medicine, IPB University, Bogor 16680, Indonesia; fhpasaribu@gmail.com; 3 Department of Livestock Production and Technology, Faculty of Animal Science, IPB University, Bogor 16680, Indonesia; astari87@apps.ipb.ac.id

**Keywords:** animal model, anserine, carnosine, lactagogue, lactation

## Abstract

Breast milk serves as the primary source of nourishment for newborns. In cases of low milk production, one approach to address this challenge involves the consumption of lactagogues. Chicken–herb essence, a beverage rich in protein, amino acids, and minerals, presents itself as a viable option to supplement a lactating mother’s diet, particularly in terms of protein intake. This study aimed to evaluate the effects of chicken–herb essence on prolactin and lactoferrin in lactating rats. Furthermore, the study also assessed the lactagogue effect on IgA in offspring. The experimental research method used a completely randomized design. The animal models in this study were female Sprague Dawley rats. The result showed that there was an increase in milk production, as seen from the results of the lactagogue effect. The highest increase in prolactin and lactoferrin was obtained in treatment group II (TG II). The increases in prolactin and lactoferrin of TG II were 214.18 ± 71.99 and 904.02 ± 435.35 pg/mL, respectively. The lactagogue test showed that TG II haspotency as a milk-booster. Testing the blood serum of offspring showed that the highest concentration of IgA was also found in TG II at 398.34 ± 214.85 pg/mL.

## 1. Introduction

Breast milk is the primary food of newborns and serves as the infant’s first immunization. It provides protection against diseases, such as respiratory infections, diarrheal diseases, and other potentially life-threatening illnesses. Exclusive breastfeeding also provides protection against obesity and certain non-communicable diseases later in life [1]. Breast milk is a fluid secreted by the mother’s mammary glands that has various benefits and properties for the baby. There are three types of breast milk produced by mothers, namely colostrum, transitional breast milk, and mature breast milk [2]. According to Boquien [3], breast milk contains 87% water, 1% protein, 4% fat, and 7% carbohydrates (including 1 to 2.4% oligosaccharides). Excluding water, the most basic components in breast milk are macronutrients, namely carbohydrates, protein, and fat [3,4,5]. Other than that, breast milk also contains many minerals and vitamins.

According to the WHO [6], as many as 136.7 million babies are born worldwide, and only 32.6% of them are exclusively breastfed for the first six months. In the Global Breastfeeding Collective, A Call to Action [7], The WHO encourages mothers to increase their early initiation commitment. The WHO also encourages mothers to exclusively breastfeed for the first six months of the baby’s life and to continue breastfeeding for up to two years or more paired up with appropriate, sufficient, and safe complementary feeding. Currently, only 39% of mothers in developing countries breastfeed exclusively.

According to the data from the Ministry of Health of the Republic of Indonesia [8], the number of mothers who breastfeed in Indonesia is still quite low, with 37.3% of mothers practicing exclusive breastfeeding, 9.3% of mothers practicing partial breastfeeding, and 3.3% of mothers practicing predominant breastfeeding. The number shown for these data is still below the national target (50%). According to the Ministry of Health of the Republic of Indonesia [8], the main reason why children from the age of 0 to 23 months were not breastfed was due to a lack of breast milk production (65.7%). Therefore, infants aged 0–5 months (33.3%), 6–11 months (35.2%), and 12–23 months (31.8%) were given pre-lacteal foods, primarily formula milk, which was the most commonly used pre-lacteal food, with percentages of 84.5%, 81.4%, and 79.9%, respectively [8]. Insufficient breast milk production is a commonly reported reason for early breastfeeding cessation, and it represents an important opportunity for intervention to improve breastfeeding outcomes [9].

One of the efforts that can be made to overcome low breast milk production is to consume foods or drugs that can increase breast milk production (lactagogue). Currently, there are several research studies being conducted on herbal ingredients that have potential as lactagogues, such as research on *Katuk* leaves (*Sauropus androgynus*), *Torbangun* leaves (*Coleus aromaticus*), and soybeans [10]. In addition to herbal ingredients, natural ingredients derived from animals also have potential as lactagogues, one of which is chicken essence. Chicken essence is a beverage that contains high protein and is rich in amino acids, minerals, and dipeptides like anserine and carnosine, which can be an alternative to help meet the mother’s intake, especially protein. Therefore, it has the potential to increase breast milk production.

Chicken essence, as a traditional beverage, has long been consumed by people to increase breast milk production. However, scientific research on chicken’s essence has not been widely studied. Currently, chicken essence is being developed with the addition of herbs to improve the sensory acceptability and to enhance the milk-booster functional effect [11]. Therefore, the researchers of this study took an interest in conducting preclinical research on the potential of chicken essence to increase milk production and infant health using Sprague Dawley rat animal models. This study aimed to analyze the effect of chicken–herb essence during lactation on prolactin, lactoferrin, and the relative weight of the mammary gland in Sprague Dawley rats to analyze the effect of chicken–herb essence during lactation.

## 2. Materials and Methods

This research obtained permission from the Animal Ethics Committee School of Veterinary Medicine and Biomedical Sciences, IPB University Number: 150/KEH/SKE/XII/2023.

### 2.1. Methods

This study was preceded by preliminary research on the production of chicken–herb essence products with ingredients consisting of a chicken carcass, red ginger, coconut sugar, sesame oil, nutmeg seeds, salt, water, and Trigona honey. The study employed native chickens, aged four months and weighing 1 kg, sourced from a poultry farm in Kemang, Bogor, Indonesia. Additional components (%*w*/*w*), including red ginger (13.72), coconut sugar (12.20), sesame oil (0.76), Trigona honey (4.23), nutmeg seeds (0.30), and salt (0.15) (Table 1), were procured from local markets and stores in Bogor. The formulation of the chicken–herb essence was adapted from a prior study [11], which employed the boiling cooking technique. The production process of the chicken essence entailed several stages: preparation, cooking, honey incorporation, and pasteurization. During the preparation phase, all food items underwent washing and weighing procedures, while the chicken was minced, and the remaining ingredients were powdered. Subsequently, in the cooking phase, all constituents except for the honey were combined and subjected to double boiling at a temperature of 100 °C for 4 h. Following this, honey was added, followed by pasteurization.

The research method used in this study was a complete randomized design consisting of four groups. These groups were the normal group, the positive control group, treatment group I, and treatment group II. The normal group was not given anything, while the positive control group was given domperidone, which is useful for increasing breast milk production [12], at a dose of 0.54 g/200 g BW. The experimental group I (TG I) received a chicken–herb essence beverage with a protein concentration of 0.162 g/200 g of the body weight of rats, while experimental group II (TG II) received a chicken–herb essence beverage with a protein concentration of 0.324 g/200 g of the body weight of rats. The characteristics of mother and offspring rats utilized in this study are shown in Table 2. The research on animal treatment and the examination of blood serum samples was conducted at the Biopharmaca Laboratory of IPB University, Laboratory of Pathology Primate Research Center IPB University, and the Pathology Laboratory of the Faculty of Medicine, Brawijaya University from March to October 2023.

The animal models in this study were female Sprague Dawley rats, and the number of samples per group was calculated according to the Federer formula (1963). The number of mothering rats required for each treatment was 6 rats. Two rats were added to each treatment group as a backup. Hence, a total of 30 rats were required. The female rats used for this research were 8–10 weeks old and weighed 150–175 g.

The types and methods of data collection. The body dimension observation data of the rats included their body weight measured using digital scales and their body length measured using a tape measure. The lactagogue effect data were obtained by measuring the milk production of the rats using either the test feeding method or the test weighing method. In this process, the mothering rats and offspring were placed in a metabolic cage and separated (weaned) for four hours to determine the full state of milk in the mammary gland then, the offspring were weighed before being reunited with the mother. The research data for this study consisted of blood serum samples obtained from the mothering rats, which were utilized for assays of prolactin and lactoferrin. These assays were conducted using an ELISA kit provided by Abbexa Ltd., Cambridge, UK [13,14]. Additionally, the offspring’s blood serum was collected at the end of the study for IgA analysis. At the final stage of the study, necropsies were performed on the mothering rats and offspring by the medical team of the Center for Biopharma Studies at IPB University. This was performed to collect blood serum for prolactin, lactoferrin, and IgA analysis.

Research stages. The study began with a ±14 day acclimation period for the rats on standard chow until the body weight of female rats reached 150–175 g and 8–10 weeks of age. Afterward, the female rats were mated with male rats (200–250 g and 8–10 weeks of age) at a 4:1 ratio and placed together in one cage. Following pregnancy, the female rats were individually placed in separate cages, with each cage containing one female rat. After birth, the total number of offspring was counted and then reduced to 6 offspring, considering the uniformity and distribution of litter size [15]. During lactation, the treatment for the mothers was fed from day 2 until day 14 of lactation when the offspring were still consuming rat milk only [15,16]. During the study, the body weights of the offspring were measured every 2 days from day 2 to day 14, and necropsy was performed on day 15. Blood samples were collected on day 14 of lactation by inducing anesthesia with 10% ketamine (dose 80 mg/kg BW) and 2% xylazine (dose 10 mg/kg BW of rats) intramuscularly until unconsciousness. The blood was collected intracardially with a 23 G syringe, then centrifuged (4000× *g*; 10 min; temperature 4 °C), and the serum was stored at −20 °C.

At the end of the treatment period, all maternal mice were sacrificed to collect mammary glands from the inguinal and thoracic regions. Subsequently, the mammary glands underwent preparation for histological analysis through hematoxylin and eosin (HE) staining, enabling the quantification of the alveolar number and measurement of diameter under a microscope at 400× magnification. The histopathological slide preparation encompassed tissue fixation using a 10% neutral buffered formalin (NBF), followed by tissue processing involving dehydration, clearing, infiltration, and embedding. Tissue samples underwent dehydration in a series of graded alcohol solutions (70%, 80%, 90%, 95%, and absolute alcohol), followed by xylene I, xylene II, and paraffin I and II immersion utilizing an autotechnicon dehydrator for a duration of 2 h. The quantification of the average number of alveoli per slide was conducted across three distinct observation areas: the tip, middle, and base. Each observation area underwent examination across 5 microscopic fields of view at 400× magnification. The measurement of the average alveolar diameter was facilitated using a microruler.

### 2.2. Statistical Analysis

The experimental data were presented as the mean ± standard deviation (SD) of the measurements and were tested for normality and processed by analysis of variance (ANOVA). In case of a significant difference between the mean values between treatments (*p* < 0.05), Duncan’s test was further conducted to see the differences between each treatment. The data obtained were analyzed using Microsoft Excel 2013 version 9.81 data processing software. The statistical analyses were performed using IBM SPSS statistical software version 22.

## 3. Results and Discussion

### 3.1. Chemical Composition

The proximate levels of the chicken–herb essence beverage are shown in Table 3, namely for carbohydrates, proteins, ash, fat, moisture, and energy. The formula contained 4.05% protein, which is a bioactive compound that is expected to increase breast milk production. The amount of chicken–herb essence given to experimental rats was based on the additional protein needs of nursing mothers of 18 g of protein [17]. This measurement was adjusted, resulting in a dose of 0.324 g/200 g BW of protein for the experimental rats.

**Figure 1:**
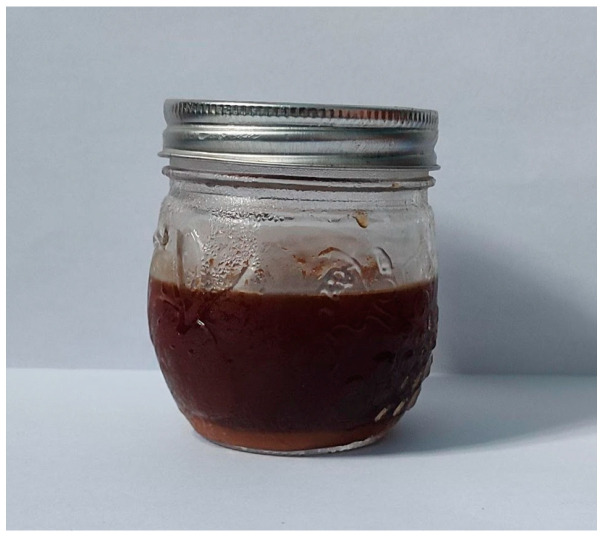
The visual appearance of chicken–herb essence.

The chicken–herb essence beverage was made from chicken carcass, red ginger, coconut sugar or palm sugar, honey, and several other ingredients, such as nutmeg, sesame oil, salt, and water. The researchers supplemented the beverage with red ginger, coconut or palm sugar, and honey, aiming to enhance the palatability of the chicken essence, which is characterized by a less desirable taste profile [18,19,20]. The main concern regarding honey is that it can contain Clostridium botulinum spores. Those spores are capable of causing infant botulinum (botulism case) in babies under 12 months old [21], whose gut flora are not yet fully developed and, thus, allow the spores to proliferate and produce botulinum toxin. However, the digestive system of a healthy adult does not support the germination of spores, and it is adept at handling these spores they are passed through our body and are excreted [22]. It efficiently processes and breaks down honey and any spores it might contain, preventing them from being transferred to the infant through breast milk. As a result, the consumption of honey does not affect the safety of breast milk, nor does it pose any risk to the baby. Visually, this beverage is presented below (Figure 1).

### 3.2. Prolactin Levels

The analysis of prolactin levels from the mother rat blood serum was taken on day 3 and day 14, the results of which can be seen in Table 4. The table showed that the difference in prolactin levels did not differ noticeably between the normal group and the treatment group I (TG I). However, the difference in prolactin levels differed noticeably in the positive control group and the treatment group II (TG II). The highest difference in prolactin levels was found in treatment group II, at 214.18 ± 71.99 mg/mL. This showed that chicken–herb essence has the potential to increase prolactin levels in accordance with the dose of the additional protein needed.

Anserine (β-alanyl-3-methyl-L-histidine) and carnosine (β-alanyl-L-histidine) are dipeptide compounds present in native chicken meat [23] and contain histidine. Histidine is known to show histamine activity, which results in a histamine-stimulating effect [24]. Since histamine accelerates prolactin secretion induced by sucking stimuli, the consumption of chicken essence promotes prolactin secretion through the stimulation of breastfeeding, which can lead to an increase in milk production [25]. The research conducted by Awano et al. [26] demonstrated the effectiveness of administering chicken extract from the 1st day of the 37th week of pregnancy until 120 h postpartum in increasing breast milk production.

The chicken–herb essence product has a better effect than pure chicken extract due to its enhanced sensory attributes from herbal ingredients. It is characterized by a reduced chicken and fishy odor, a sweeter, sour, and savory taste, and a less bitter flavor [11]. Furthermore, the herbal ingredients provide benefits; for example, red ginger contains an active proteolytic enzyme known as zingibain, which can hydrolyze the peptide bond in meat. Additionally, red ginger is also an aromatic spice with a fresh aroma that can prevent a fishy smell [27].

### 3.3. Lactoferrin Levels

The analysis of lactoferrin levels in the blood serum of parent rats was carried out similarly to the prolactin testing. Blood serum was taken on day 3 and day 14 to observe any changes in lactoferrin levels during lactation. The result of this test can be seen in Table 5, which shows the lactoferrin levels and differences on day 3 and day 14. The highest lactoferrin level on day 3 was found in the positive control group of 1590.13 ± 292.43 pg/mL, while the highest level on day 14 was found in the treatment group II of 1961.27 ± 366.09 pg/mL. The greatest difference in lactoferrin was found in treatment group II, measuring 904.02 ± 435.35 pg/mL. This shows that treatment group II experienced the greatest increase in the concentration among other treatment groups.

Lactoferrin, the second most abundant protein in human milk, is a multifunctional protein produced in the epithelium of mammary glands and subsequently secreted into milk [28,29]. Upon the consumption of breast milk, lactoferrin offers early-life immune protection to infants [30]. Comprising over 600 amino acids, lactoferrin exhibits a myriad of activities in breast milk, including antibacterial, antifungal, antiviral, antiparasitic, anti-inflammatory, and immunomodulatory effects [31]. Its primary function involves binding iron in the digestive tract, thereby attenuating the proliferation of pathogenic bacteria reliant on iron for growth [32].

### 3.4. Immunoglobulin A

Table 6 shows the results of IgA levels in offspring. The table indicates that chicken–herb essence beverages affect the IgA levels of offspring. Duncan’s tests further show that IgA levels in the normal group, positive control group, and treatment group I were not significantly different. However, treatment group II was significantly different from the other mothering rats with a protein dose of 0.324 g/200 g BW.

IgA is the main immunoglobulin in breast milk. The synthesis of SIgA during lactation increases and then decreases in the period of mature breast milk [30]. Table 4 shows higher IgA levels in treatment group II, indicating that giving herbal chicken essence beverages has the potential to increase maternal milk production. When producing more milk, the baby receives more SIgA. Therefore, the levels of IgA received by the baby during the breastfeeding process were also increased [30].

### 3.5. Lactagogue Test

**Figure 2:**
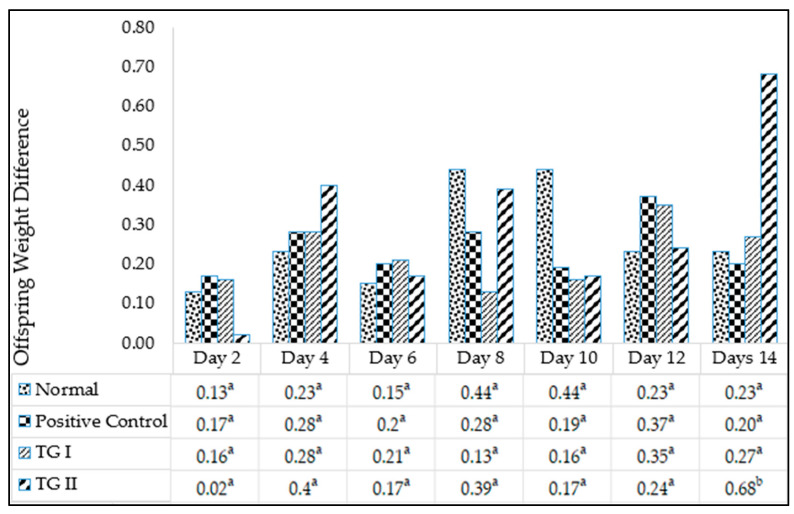
Lactagogue test on Sprague Dawley rats. ^a,b^ Different superscript letters in each column are significantly different at *p* < 0.05.

The lactagogue test results are depicted in Figure 2. The figure illustrates that the highest weight gain occurred on day 14 in treatment group II, showing a difference in baby rat weight of 0.68 units. Substances exhibiting a lactagogue effect typically lead to an increase in milk production or volume secretion [33]. Consequently, it can be inferred that the herbal chicken essence beverage influences the weight gain of baby rats and exhibits a lactagogue effect.

The lactagogue test serves as a means to quantify the milk produced by rats, utilizing either the test-feeding or test-weighing method. This measurement entails assessing the variance in infant weight before and after breastfeeding to determine the quantity of milk consumed [34]. Given the challenge of directly measuring milk production in rats, this method is deemed acceptable for evaluating sufficient milk production [35].

### 3.6. Alveolar Cells in Mammary Gland

**Figure 3:**
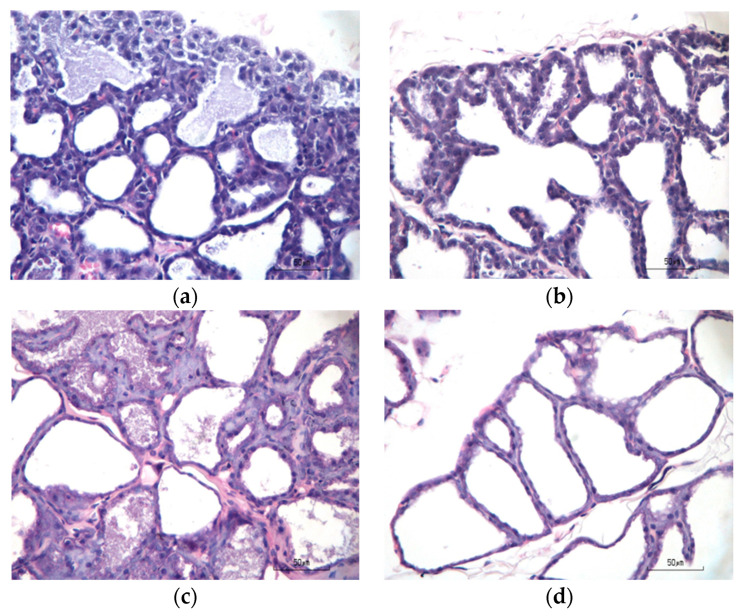
The histopathological examination of mammary gland tissues involved staining panels (**a**–**d**) with hematoxylin and eosin (H&E, 400× magnification). The number of alveolar cells per field of view. (**a**) Normal; (**b**) positive control; (**c**) treatment Group I; and (**d**) treatment Group II.

Alveolar cells in the mammary gland for four treatment groups are shown in Figure 3. Data collection for this histopathological analysis was performed on a subsample basis, with three subjects selected from each treatment group. Alveolar cells were extracted from both the inguinal and thoracic regions, and microscopic observations were conducted to enumerate the number of alveolar cells per field of view; each observation area underwent examination across five microscopic fields of view, measuring the diameter of these cells.

**Figure 4:**
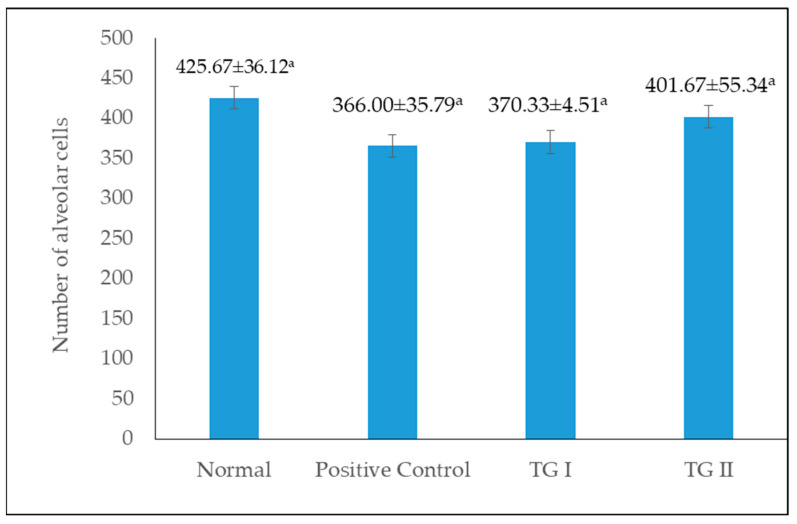
Number of alveolar cells per alveoli in the mammary gland. Same superscript letters in different group treatments are not significantly different (*p* > 0.05).

Figure 4 shows the number of alveoli cells in the normal, positive control, TG I, and TG II groups. The highest number of cells was found in the normal group, but among the intervention groups (positive control, TG I, and TG II), the highest number of alveolar cells were found in the TG II group. In this study, there was no significant difference in the number of alveolar cells among all treatment groups. However, in the groups given domperidone and chicken–herb essence intervention (positive control, TG I, and TG II groups), the highest number of alveoli was observed in the TG II group, indicating that the administration of chicken–herb essence tends to increase the number of alveolar cells.

**Figure 5:**
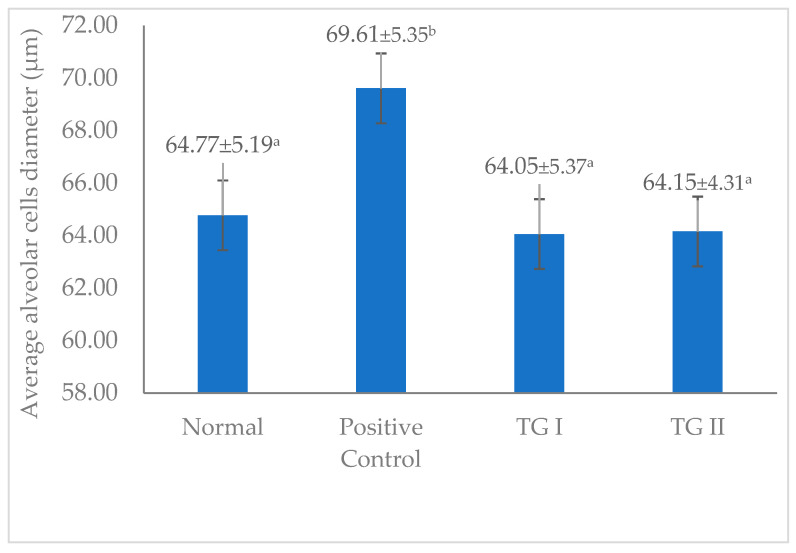
Average alveolar cell diameter in the mammary gland. ^a–b^, Different superscript letters in each column are significantly different at *p* < 0.05 and analyzed using one-way ANOVA and Duncan’s post hoc test. TG I, treatment group I; TG II, treatment group II.

Figure 5 shows the average alveolar cell diameter in the normal, positive control, TG I, and TG II groups. The figure shows that the difference in alveolar cell diameter did not differ noticeably between the normal group, TG I and TG II. However, the difference in alveolar cell diameter differed noticeably in the positive control group. In this study, the positive control group administered domperidone exhibited the greatest average diameter of alveolar cells. Domperidone is one of the drugs used in many countries (Australia, Belgium, Canada, Ireland, Italy, Japan, the Netherlands, and the United Kingdom) as a galactagogue (off-label) [29]. Domperidone, as an antidopaminergic, has the potential to increase prolactin concentration and milk production and enhance lactation [36,37].

Milk production is facilitated by the epithelial cells within the mammary glands of lactating mammals. The ability of the mammary gland to generate milk significantly relies on the quantity and functionality of these mammary epithelial cells [38]. The alveoli are dilated at the condition of the lactating mammary glands, and the walls separating the various alveoli can be seen clearly. The alveolar acts as sacs for milk production. The wider the alveolar and the more lobes in the mammary glands during the lactation period, the greater the milk production. Alveoli consists of epithelial cells with high proliferative capacity. During lactation, nursing activity is highly active, and milk production causes the alveolar lumen to enlarge; the secretory activities of the cells within the alveoli result in the secretion of milk components into the alveolar lumen [39]. In the lactating mammary gland, there is a significant expansion of blood vessels within the stroma to support milk production by providing a large number of micro and macromolecules [40].

### 3.7. Lymphocyte Cells in Mammary Gland

**Figure 6:**
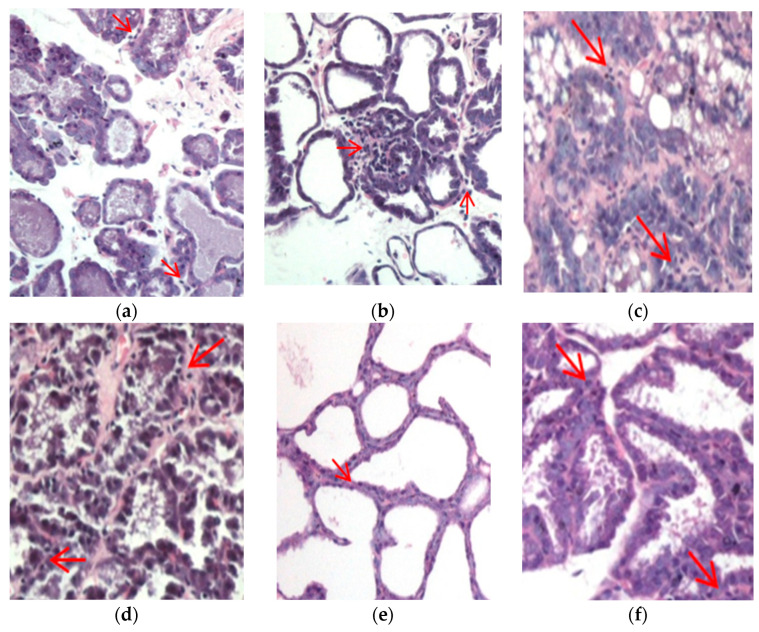
Lymphocyte cells in mammary glands. (**a**) Observation of lymphocyte cells in the positive control group of the inguinal region; (**b**) observation of lymphocyte cells in the positive control group of the thoracic region; (**c**) observation of lymphocyte cells in treatment group I in the inguinal region; (**d**) observation of lymphocyte cells in treatment group I in the thoracic region; (**e**) observation of lymphocyte cells in treatment group II in the inguinal region; and (**f**) observation of lymphocyte cells in treatment group II in the inguinal region. Red arrow shows the presence of lymphocyte cells in the mammary gland.

The analysis of lymphocyte cells in the mammary gland can be seen in Figure 6. In this study, we observed the presence of lymphocyte cells in the mammary gland across all treatment groups. Except for the normal group, lymphocyte cells were assessed based on their presence in the entire field of view.

Lymphocytes, as a type of inflammatory cell, were observed in the mammary gland tissue. Based on observations across the entire field of view of the mammary gland tissue, the presence of lymphocytes in the positive control group ranged from 0 to <25%, while in treatment group I, the presence of lymphocytes ranged from 25 to 50%. In treatment group II, the presence of lymphocytes ranged from 0 to <25%. Lymphocytes play a role in optimizing the immune system’s function. Immune cells constitute a natural component of the mammary gland stroma throughout various stages of postnatal mammary gland development [41]. The presence of immune cell accumulation in mammary glands supports the notion that the mammary gland is capable of eliciting a genuine local immune response [42]. However, to which compartment of the mammary gland these lymphocytes migrate remains largely unknown [42]. Lymphocytes that enter the milk must cross the ductal basement membrane and penetrate the layer of luminal epithelial cells [43]. Throughout this process, luminal epithelial cells produce chemoattractants that guide the migration of immune cells. Certain chemokines, such as CCL2 and CCL28, facilitate the transmigration of lymphocytes [42]. CCL28 assists in the migration of cells through the vascular endothelium into tissues like the skin and breast [44] and the presence of CCL2 in milk, which likely plays a significant role in attracting immune cells [45]. Notably, research by Dill and Walker [46] has shown that prolactin boosts the production of chemoattractants, affecting luminal MECs. However, little is known about how hormones promote lymphocyte migration by adjusting chemokine levels. The number and proportion of these cells in mammary secretions have varied in some studies, which could reflect the physiological and immunological conditions [42]. Lymphocytes within the mammary gland are pivotal constituents of colostral immunity. Studies have indicated that 83% of lymphocytes present in milk are T cells, while 6% are B cells [47,48]. Therefore, our study suggests that chicken–herb essence may activate the immune system during lactation.

The advantages of this study were that we observed alveolar cells in mammary glands through a histopathological study conducted on experimental animals. The limitations of this study are that we could not further analyze the types of bioactive compounds in chicken–herb essence, and the obtained result could not be easily extrapolated from animal models to humans therefore, further studies are necessary to explore the potential of chicken–herb essence in humans.

## 4. Conclusions

This study concludes that there was an observed increase in breast milk production, as seen from the results of the lactagogue effect. Notably, the results of prolactin and lactoferrin tests in the mothering rat blood serum were highest in treatment group II. Furthermore, testing the lactagogue effect in offspring revealed that TG II has a lactagogue effect. The highest concentration of IgA was also found in TG II. Therefore, chicken–herb essence beverages have the potential to increase breast milk production (lactagogue). Suggestions for further research include analyzing concentrations of bioactive dipeptide compounds (anserine and carnosine) to increase breast milk production and analyzing the effect of chicken–herb essence on other parameters, such as IGF or TGF hormones, which play a role in lactation and infant health.

## Figures and Tables

**Table 1:** The development of a testing formula.

Ingredients	Testing Formula (g, %)
Chicken carcass	800 (45.75)
Red ginger	240 (13.72)
Coconut sugar	213.3 (12.20)
Sesame oil	13.3 (0.76)
Nutmeg seeds	5.3 (0.30)
Salt	2.7 (0.15)
Trigona honey	74 (4.23)
Water	400 (22.88)

**Table 2:** The characteristics of lactation and offspring rats utilized in the in vivo study of milk-boosting chicken–herb essence.

Animal Code	Treatment Group	Weight (g)		Number of Offspring	Cholesterol (mg/dL) (Day-14)	Protein Total (g/dL) (Day-14)
		Day-2	Day-14			
001	Normal	224	245	6	72.69	6.5
002	Normal	257	257	6	50.60	6.1
003	Normal	223	195	6	33.44	6.2
004	Normal	207	189	6	44.35	6.7
005	Normal	234	245	6	81.23	5.9
006	Normal	202	205	6	67.66	6.6
007	Positive control	193	181	6	49.42	5.2
008	Positive control	228	235	6	56.59	6.0
009	Positive control	228	227	6	58.31	6.2
010	Positive control	210	185	6	40.86	6.3
011	Positive control	190	189	6	44.49	5.7
012	Positive control	190	173	6	37.92	6.4
013	TG I	219	194	6	89.99	6.4
014	TG I	225	200	6	107.49	6.1
015	TG I	174	173	6	101.15	6.3
016	TG I	168	164	6	61.94	6.3
017	TG I	221	230	6	60.31	6.5
018	TG I	251	247	6	64.12	6.9
019	TG II	230	217	6	52.89	6.5
020	TG II	175	175	6	60.76	6.4
021	TG II	195	180	6	51.16	6.6
022	TG II	205	211	6	70.97	6.3
023	TG II	229	246	6	60.53	5.4
024	TG II	222	220	6	38.41	6.3
Mean ± SD ^1^		212.50 ± 23.18	207.63 ± 28.43	6	60.72 ± 19.23	6.24 ± 0.39

^1^ SD, standard deviation.

**Table 3:** Chemical composition content of chicken–herb essence. wb, wet basis.

Chemical Composition	Unit	Amount
Carbohydrate Protein	%wb	26.39 ± 2.78
	%wb	4.05 ± 0.26
Fat	%wb	<0.02
Ash	%wb	0.73 ± 0.04
Moisture	%wb	68.84 ± 2.96
Energy	kcal/100g	121.75 ± 11.68
Dipeptide	ng/mL	353.52 ± 19.71

**Table 4:** Effect of chicken–herb essence beverage on prolactin levels.

Treatment Groups	Prolactin Day 3 (mg/mL)	Prolactin Day 14 (mg/mL)	Prolactin Level over 11 Days (mg/mL)
Normal	87.11 ± 50.92 ^a^	72.99 ± 34.35 ^a^	−14.12 ± 70.40 ^a^
Positive control	212.02 ± 25.12 ^b^	79.19 ± 23.65 ^a^	−132.82 ± 15.30 ^b^
TG I	71.76 ± 15.50 ^a^	63.17 ± 11.47 ^a^	−8.59 ± 21.98 ^a^
TG II	52.25 ± 8.49 ^a^	266.43 ± 69.86 ^b^	214.18 ± 71.99 ^c^

^a–c^, Different superscript letters in each column are significantly different at *p* < 0.05 and analyzed using one-way ANOVA and Duncan’s post hoc test. TG I, treatment group I; TG II, treatment group II.

**Table 5:** Effect of chicken–herb essence beverage on lactoferrin levels.

Treatment Groups	Lactoferrin Day 3	Lactoferrin Day 14	Lactoferrin Level over 11 Days
	(pg/mL)	(pg/mL)	(pg/mL)
Normal	1122.62 ± 411.57 ^a^	1342.32± 123.25 ^a^	219.70 ± 503.10 ^a,b^
Positive control	1590.13 ± 292.43 ^b^	1360.67 ± 143.51 ^a^	−229.47 ± 290.37 ^a^
TG I	880.95 ± 137.17 ^a^	1427.95 ± 122.82 ^a^	547.00 ± 226.37 ^b,c^
TG II	1057.25 ± 141.19 ^a^	1961.27 ± 366.09 ^b^	904.02 ± 435.35 ^c^

^a–c^, Different superscript letters in each column are significantly different at *p* < 0.05 and analyzed using one-way ANOVA and Duncan’s post hoc test. TG I, treatment group I; TG II, treatment group II.

**Table 6:** Effect of chicken–herb essence beverage on IgA levels of offspring.

Treatment Groups	IgA (pg/mL)
Normal	201.91 ± 31.94 ^a^
Positive control	200.08 ± 22.66 ^a^
TG I	231.57 ± 92.33 ^a^
TG II	398.34 ± 214.85 ^b^

^a,b^, Different superscript letters in each column are significantly different at *p* < 0.05 and analyzed using one-way ANOVA and Duncan’s post hoc test. TG I, treatment group I; TG II, treatment group II.

## Data Availability

The original contributions presented in the study are included in the article, further inquiries can be directed to the corresponding author.
